# Repetitive seizures after febrile period exclusively involving bilateral claustrum

**DOI:** 10.1097/MD.0000000000027129

**Published:** 2021-09-17

**Authors:** Fan Yang, Lichao Sun, Jing Li, Weihong Lin

**Affiliations:** a Department of Neurology, The First Hospital of Jilin University, Changchun, Jilin, China; b Department of Emergency, The First Hospital of Jilin University, Changchun, Jilin, China.

**Keywords:** claustrum, febrile, magnetic resonance images, seizure

## Abstract

The purpose of this study is to demonstrate the relationship between acute repetitive seizures and claustrum damage, and to provide basis for the treatment of repetitive seizures exclusively involved the bilateral claustrum.

Between August 2014 and October 2015, 5 patients with repetitive seizures after a febrile period were admitted to our hospital, showing exclusive involvement of bilateral claustrum on magnetic resonance images (MRI). All patients underwent serum virology testing, autoimmune antibody test, MRI, and electroencephalograph examination.

All patients were young women (16–29 years) with an unremarkable previous medical history, and 2 of them were pregnant. Similar clinical symptoms like antecedent febrile illness in the 3 to 7 days preceding seizures, psychiatric disorder, or dysautonomia occurred in 5 patients. Abnormal MRI signals exclusively confined to the bilateral claustrum appeared in 4 patients during the acute phase and in 1 patient during the chronic phase. All patients accepted empirical treatment with anti-viral and anti-seizure drugs and had good outcomes (seizure-free, though with some residual short-term memory loss) at the 3rd year follow-up.

Although the clinical and associated brain imaging findings were characteristic, the etiology was still unclear. Contrary to previous studies, the patients presented here have all received a good prognosis.

## 1 Introduction

Repetitive seizures after a febrile period, a common clinical symptom, affects 1 in 1,000,000 children but also occurs in adults.^[1–3]^ It is often caused by central nervous system encephalitis (viruses, bacterial, immune-mediated, etc). With the development of immunology and people's understanding of encephalitis, autoimmune encephalitis can be more readily distinguished from viral encephalitis.^[4,5]^ However, there are still 40% to 60% encephalitis whose etiology is still unclear clinically.^[6–9]^ In response to this, a consensus definition of febrile infection-related epilepsy syndrome (FIRES) applicable to all ages was suggested at the First International new-onset refractory status epilepticus and FIRES symposium.^[10]^ The new definition for FIRES is that it requires a prior febrile infection, with fever starting between 2 weeks and 24 hours prior to the onset of refractory status epilepticus, with or without fever at onset of status epilepticus (SE). According to the clinical course, FIRES comprises as fever period, acute and chronic phases.^[11–13]^ A poor outcome of FIRES was reported in many studies.^[14–18]^ With the definition emergence, some patients with SE after fever period can be summarized. Patients having recurrent seizures after fever without SE could not be classified as FIRES.

The claustrum is a thin, irregular sheet of grey matter on both hemispheres, concealed between the insula and putamen.^[19,20]^ Its anatomical structure is quite remarkable in that it receives input fibers from almost all regions of the cerebral cortex and projects back to almost all regions of the cortex.^[20]^ In recent years, the claustrum was thought to play a key role in sensory integration and consciousness.^[20–23]^

Here we report 5 young women cases with repetitive seizures after a febrile period, and 2 of 5 cases were in accordance with the definition of FIRES. In these cases, bilateral claustrums were involved exclusively and outcomes were surprising. As far as we know, this is the first retrospective study from China showing these results.

## 2 Methods

**Figure 1:**
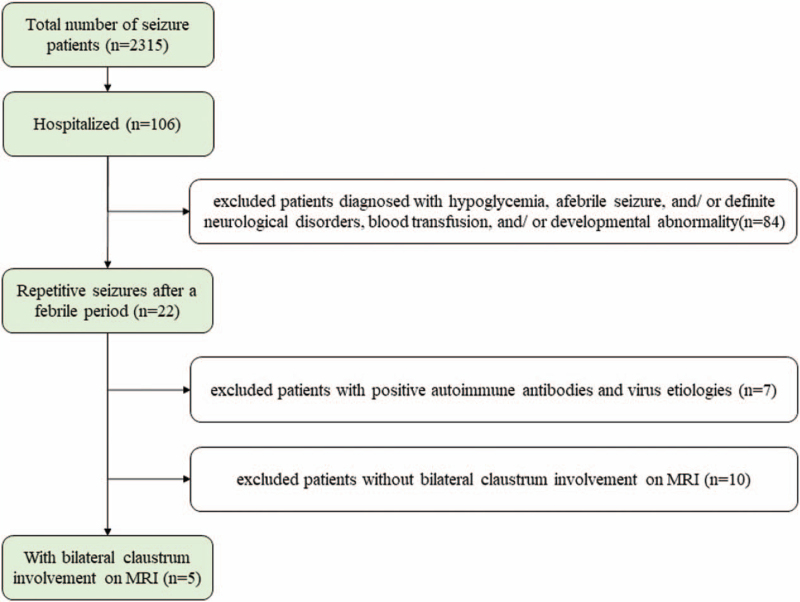
The experimental flow chart. Only 5 patients were involved.

We conducted a retrospective study on all patients with repetitive seizures after febrile periods admitted to the First Hospital of Jilin University from August 2014 to October 2015. All patient data were identified using electronic medical records. We included a total of 5 patients with repetitive seizures after febrile periods, which were characterized by exclusive claustrum involvement on MRI (Fig. 1). We excluded patients diagnosed with hypoglycemia, afebrile seizure, and/ or definite neurological disorders, blood transfusion, and/ or developmental abnormality. We further excluded patients with positive autoimmune antibodies and virus etiologies. Their medical history, clinical symptoms, laboratory tests, imaging, treatment, and follow-ups were summarized and analyzed in this study.

The TORCH test (including Toxoplasma gondii, rubella virus, cytomegalovirus, herpes simplex virus) of the serum and cerebrospinal fluid (CSF) were carried out for virus screening and the records of virus levels were expressed by signal to the cut-off ratio. The CSF was examined for physical properties, conventional, biochemical parameters, and viral etiologies. Both serum and CSF were sent to Peking Union Medical College Hospital for autoimmune antibodies screening, including neuropil antibodies against N-methyl-D-aspartate receptor, α-amino-3-hydroxy-5-methyl-4-isoxazole propionic acid receptor type 1 and type 2, contactin-associated protein-2, leucine-rich glioma inactivated-1, and gamma-aminobutyric acid-B. The screening was also performed for anti-neuronal autoantibodies such as anti-amphiphysin, anti-CV2, anti-PNMA2 (Ma2/Ta), anti-Ri, anti-Yo, and anti-Hu.

Brain MRI (1.5T) results were reviewed in detail by a board-certified neuroradiologist who examined T1-weighted images, T2-weighted images, and fluid-attenuated inversion recovery images (with water and lipid suppression). Twenty-lead electroencephalograph (EEG) with video monitoring for 24 hours was underwent on all patients.

The patients were subsequently treated with a combination of antiviral drugs and anti-seizure drugs (ASDs) and followed up by telephone after leaving the hospital. The patients were instructed to go to the hospital to adjust treatments in case of seizure recurrence.

## 3 Results

### 3.1 Patients’ clinical characteristics

All patients were women with a mean age of 22.8 years (range: 16–29 years). There were 2 cases of pregnancy and 2 cases of cardiac history, which resulted in a rather complicated treatment. In addition, 1 patient had twice seizures after shock at the age of 3, but denied the history of fever seizure and recurrence before this infection. All patients presented with prodromal infection symptoms, including headache and fever with a body temperature between 38 and 41 °C. The seizure types included generalized tonic-clonic seizure in 3 out of 5 patients, and focal motor seizure with involuntary lip-smacking in 2 out of 5 patients. We observed that 3 patients without SE had an average of 4.6 ictal events before and during hospitalization, ranging from 3 to 6 seizures, and the duration was 1 to 5 minutes. Status epilepticus was the first manifestation in 2 patients, and the first seizure occurred 3 to 7 days after the febrile illnesses. We noticed more than 13 and 7 ictal events, respectively, lasting up to 20 minutes.

Four patients showed psychiatric disorders, including memory loss (3/4), garrulousness (3/4), agitation, and nonsense speech. Symptoms associated with dysautonomia were also reported in our study, including chest distress, heart palpitation, shortness of breath (4/5), vomiting (2/5), and respiratory arrest (1/5). Table 1 summarizes the main clinical manifestations of the patients.

**Table 1:** Major clinical characteristics of 5 patients at admission.

Case No.	Age, y	Pregnancy	Past medical history	Fever	Headache	Symptoms of psychiatric disorder	Seizure type	Seizure count	Memory loss	Automatic instability
1	16	–	Patent foramen ovale	+	–	Garrulousness, agitation	GTCS	5	+	Chest distress, heart palpitation, shortness of breath, and blue lips
2	26	–	Myocarditis	+	+	Garrulousness, agitation, nonsense speech,	FMS	6	+	Heart palpitation
3	29	+ (for 1 month)	–	+	+	Garrulousness, agitation, nonsense speech	GTCS	3	–	Heart palpitation, shortness of breath, lip paresthesia, and vomiting
4	20	+ (for 4 months)	Seizure	+	–	–	SE, GTCS	More than13	–	Vomiting
5	23	–	–	+	–	agitation	SE, FMS	More than 7	+	Respiratory inhibition and heart palpitation

FMS = focal motor seizure, GTCS = generalized tonic-clonic seizure, SE = status epilepticu.

### 3.2 Laboratory examination

Three of them were negative for the TORCH test Immunoglobulin G (IgG). Anti-herpes simplex virus (HSV) IgG (+), anti-rubella virus IgG (+), and anti-cytomegalovirus IgG (+) were increased in the other 2 patients, while anti-toxoplasma gondii IgG was negative. All patients were negative for Immunoglobulin M (IgM), excluded the recent infection of TORCH viruses. The viral etiologies of CSF were negative in 5 patients.

The CSF of all patients was clear, and the puncture pressure was between 145 and 270 mmH2O. As shown in Table 2, 3 patients had pleocytosis (white blood cell count to >5 cells per mm^3^) with monocytes accounted for 90% of the total white blood cells, and 1 had an increased protein concentration. Oligoclonal bands in the CSF were not tested. The examined autoimmune antibodies in serum and CSF of all patients were negative.

**Table 2:** Conventional and biochemical parameters of CSF.

				White blood cell composition		
Case No.	Protein, g/L	Glucose, mmol/L	White blood cell count, ×10^6^/L	Multinuclear leukocyte (%)	Monocyte (%)	IgG (S/CO)
1	0.29	3.42	33	0.1	0.9	18.7
2	0.5	5.82	33	0.1	0.9	42.8
3	0.45	3.35	5	–	–	
4	0.22	3.9	3	–	–	10.0
5	0.26	5.68	9	0.1	0.9	19.7

CSF = cerebrospinal fluid, S/CO = cut-off ratio.

### 3.3 MRI and EEG examination

**Figure 2:**
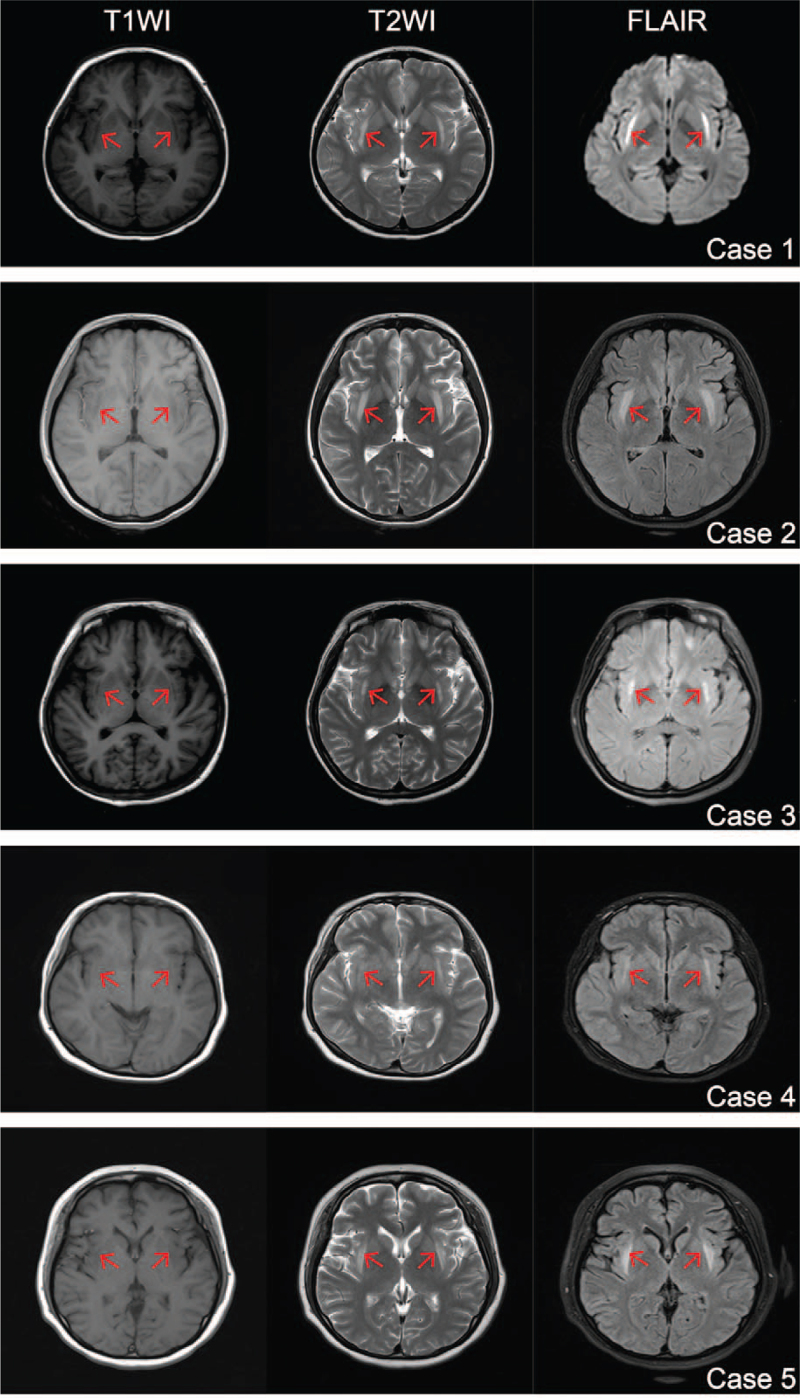
Magnetic resonance images of 5 cases. The claustrum of all patients showed symmetrical low signal lesions on T1WI, high signal lesions on T2WI, and FLAIR (red arrows). FLAIR = fluid-attenuated inversion recovery, T1WI = T1-weighted images, T2WI = T2-weighted images.

Figure 2 shows representative cerebral MRI results. The claustrum of all patients had symmetrical hypointense lesions on T1-weighted images (1A–5A), hyperintense lesions on T2-weighted images (1B–5B), and fluid-attenuated inversion recovery (1C–5C). These abnormal signals showed up within a week after the initial seizure in 4 patients. Because the other patient was in a serious condition, the imaging was acquired at the chronic phase (19 days from SE onset).

**Figure 3:**
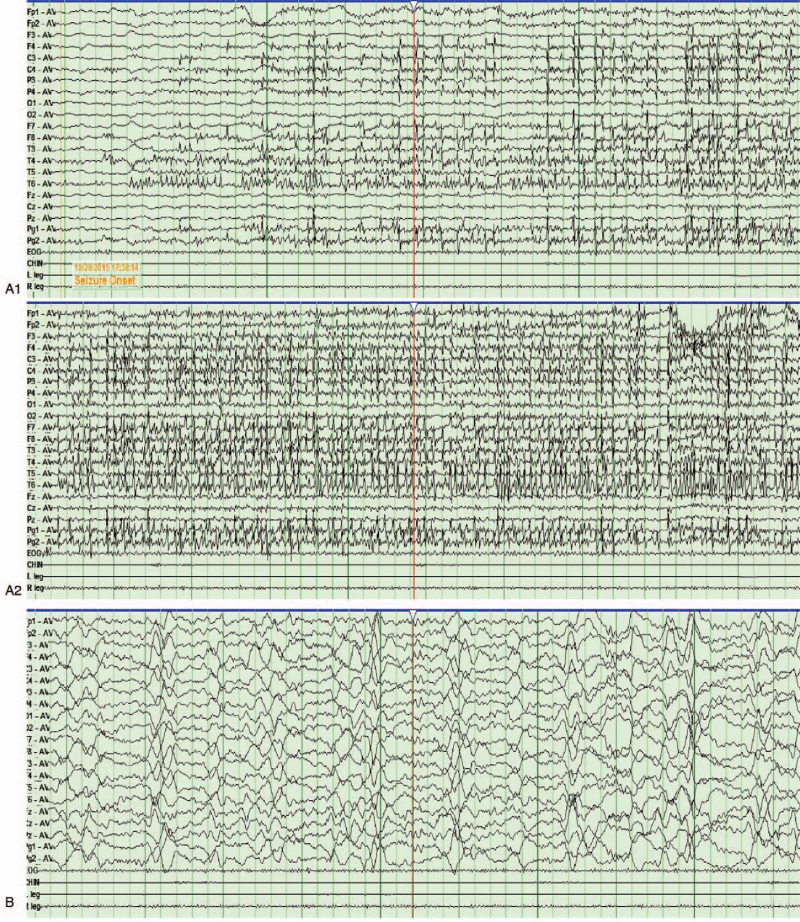
EEG recording of patient 5. (A) In the acute phase (day 13) showing a focal motor seizure with a fast ictal rhythm arising from the left temporal region. Intraictal activation of the right temporal region is seen. (B) In interictal, multifocal irregular sharp and slow waves of medium-high amplitudes were observed in the bilateral temporal and frontal regions. EEG recording parameters: low frequency = 1 Hz, high frequency = 70 Hz, normal frequency = 50 Hz, sensitivity = 10 μV, and chart speed = 30 mm/s. EEG = electroencephalograph.

As detected by 24 hours EEG, all patients showed sharp and slow wave complex with irregular frequencies in frontal and temporal lobes. SE was observed in 2 patients. Figure 3 shows the typical manifestation of seizures in 1 patient.

### 3.4 Treatment and outcome

The average hospitalization time was 26.4 days. Considering that the anti-viral treatment may affect fetal development, the 2 pregnant patients voluntarily aborted, striving for better treatment and signing informed consent. Based on the above-described clinical presentations and emergency examination results, encephalitis should be the prime differential diagnosis, thus a tentative diagnosis of suspected viral encephalitis was made in all patients. All patients were treated with acyclovir (0.5 g per 8 hours), an anti-viral drug. Depending on the severity of seizure, all patients were treated with ASD (monotherapy or polytherapy). No patient was administered with steroids or immunoglobulin in our study.

Two patients showed SE after the febrile periods were treated with diazepam (20 mg, intravenous injection) and pentobarbital (0.2 g, intramuscular injection) as initial therapy. As the duration of epileptic seizure was 20 minutes, continuous intravenous infusion of sodium valproate (2 mL/h) was used as second-line therapy. In these 2 cases, one continued to receive this second-line treatment due to an ongoing seizure. Another had seizures with asphyxia 10 days before admission to our hospital. She received emergency tracheal intubation and assisted mechanical ventilation in another hospital. Therefore, we applied the third-line therapy, continuous intravenous infusion of propofol (2 mL/h). Their SE was controlled after 7-days’ treatment. When patients only had cluster seizures, we chose oxcarbazepine (3/5), levetiracetam (5/5), sodium valproate (2/5), and carbamazepine (1/5) for monotherapy or polytherapy. Furthermore, 1 patient with serious psychiatric disorders was administered with clonazepam and risperidone under the guidance of a psychiatrist.

All patients showed improvement in clinical symptoms after 10 to 15 days of treatment. The seizures were reduced, the psychiatric symptoms were alleviated, but the memory lost still. During the 3 years’ follow-up, 2 patients still suffered monthly seizures, and 1 patient had seizure attacks when irregular administration or drugs change in the first year. All patients were seizure-free and gradually reduced ASDs in the third year. Nevertheless, 4 of the 5 patients presented residual short-term memory loss.

## 4 Discussion

We report a homogenous group of 5 young female patients with repetitive seizures after febrile periods. Two out of 5 presented with SE as the first manifestation. They all responded positively to anti-seizure and anti-viral treatment. The brain imaging findings in all patients exhibited abnormal signals exclusively confined to the bilateral claustrum. No evidence of central nervous system infection was found.

The claustrum, a telencephalic and subcortical structure, is located deep medial to the insula and superficial to the basal ganglia.^[24]^ It plays a key role in integrating diverse sources of neural information during the formation of unified conscious percepts.^[20,21]^ Claustro-cortical fibers connect the claustrum with several cortical areas, including the prefrontal, prepostcentral, posterior parietal, orbitofrontal, and medial temporal cortex.^[23,25,26]^ Moreover, electrical stimulation studies have evoked multimodal responses from the insula.^[27]^ Therefore, we think it could explain why our study is slightly different from previous studies. The claustro-cortical fibers damage may involve the insula and its connected regions. As a result, our patients suffered from autonomic instability (chest distress, heart palpitation, shortness of breath, vomiting, and respiratory arrest), memory loss, garrulousness, agitation, and nonsense speech. Instead, the reported common symptoms, in previous studies, include vomiting, lethargy, fluctuation of vigilance, stupor, seizures, and a variety of auras (somatosensory, psychic, auditory, “out of body”).^[18,28]^

Encephalitis as the prime differential diagnosis should be considered first. At present, the etiological diagnosis of encephalitis remains difficult, due to the limitations of current knowledge and examination techniques. Viral encephalitis is the major cause of acute neurologic damage and disability in children worldwide.^[29]^ More than 130 viruses are able to induce encephalitis, including human enterovirus, herpes simplex virus types 1 and 2, varicella, varicella-zoster virus, Japanese B encephalitis virus, cytomegalovirus, mumps virus, and rubella virus. Nevertheless, up to 60% of the presumed viral encephalitis cases remain unexplained due to the failure of conventional laboratory techniques to detect an infectious agent.^[30]^ Three cases of bilateral symmetrical bilateral claustrum lesions caused by viral infection have been reported, including 2 cases of HSV,^[31,32]^ and 1 case of mumps virus.^[33]^ In our study, IgM of HSV and the other uncommon virus was not tested.

In recent years, breakthroughs have been made in the understanding of autoimmune diseases and autoimmune antibodies, greatly expanding the scope of clinical diagnosis of encephalitis.^[34]^ Hiraga A described a 65-year-old female patient with voltage-gated potassium channel antibodies and limbic encephalitis in 2014, whose brain MRI showed symmetrical bilateral medial temporal lobe and claustrum lesions.^[35]^ Striking bilateral claustrum lesions were reported in 4 patients with autoimmune epilepsy in 2007.^[28]^ Whereas, we did not find any autoimmune antibodies leading to the damage in our patients.

Many studies on adult FIRES/new-onset refractory status epilepticus were reported from 1994 to 2018, but there was no identifiable cause.^[18,36–39]^ Brain radiographic change of children with FIRES can be found in temporal lobe, insula, hippocampus, thalamus, and basal ganglia.^[14,15,16,17,40]^ Damages, especially those involving the claustrum, were rarely summarized.^[18,36]^ In Meletti et al^[36]^ cases, fever preceded SE in 28 out of 31 patients, with an average of 6 days. In addition, all patients showed claustrum changes on MRI at 10 days after SE on average. They suggested that, claustrum may play a relevant role in maintaining an aggressive refractory pattern of SE, although the sequence of SE and claustrum damage cannot be determined. Since claustrum is widely connected with multiple cortical regions and consists of densely packed and closely connected GABAergic interneurons, some previous studies have also supported the view that claustrum is a key region participating in the development of generalized seizures.^[20,23,25,41,42]^ Based on the algorithm for the diagnosis of autoimmune encephalitis suggested by Graus et al in 2016^[43]^ and the definition of FIRES,^[10]^ 2 of our cases with SE satisfied the description of the latter. However, the definition of FIRES is not sufficient to generalize the recurrent seizures after a febrile event, leading to the exclusion of this diagnosis in three fifths of patients in our study.

The cases mentioned in the previous 3 paragraphs were characterized by epileptic seizures or SE due to different etiologies. Most patients continued to have intractable epilepsy. Interestingly, our patients all displayed good outcomes after anti-seizure and anti-viral treatment. The discrimination suggests that the etiology may be different from the previous ones. Our study shows that the count of seizure events in in patients without SE is much less than that in SE patients, which may be improve the prognosis to a certain extent. Unfortunately, we didn’t obtain new MRI results, which might be a better indication of the improvement in the patients’ condition. Besides, the efficacy of immunotherapy, which usually applied to encephalitic patients, also needs to be proven by follow-up clinical and experimental studies.

In conclusion, we have reported that 5 young women had repetitive seizures after febrile periods with exclusive bilateral claustrum lesions. Although we speculate that these cases further demonstrate the correlation between claustrum damage and acute repetitive seizures, much more work is needed to verify the relationship and the etiology of these cases.

## Acknowledgments

The authors sincerely thank all the participants in this project.

## Author contributions

Fan Yang and Jing Li collected the data. Lichao Sun and Weihong Lin designed and wrote the manuscript. All authors read and approved the final manuscript.

**Conceptualization:** Lichao Sun, Jing Li.

**Data curation:** Lichao Sun.

**Formal analysis:** Fan Yang.

**Funding acquisition:** Weihong Lin.

**Project administration:** Weihong Lin.

**Writing – original draft:** Jing Li.

**Writing – review & editing:** Fan Yang, Jing Li.
